# Femtosecond Laser-Induced Phase Transformation on Single-Crystal 6H-SiC

**DOI:** 10.3390/mi15020242

**Published:** 2024-02-06

**Authors:** Hongsheng Quan, Ruishi Wang, Weifeng Ma, Zhonghuai Wu, Lirong Qiu, Kemi Xu, Weiqian Zhao

**Affiliations:** The MIIT Key Laboratory of Complex-Field Intelligent Exploration, School of Optics and Photonics, Beijing Institute of Technology, Beijing 100081, China; quanhongsheng@163.com (H.Q.); wangrs@bit.edu.cn (R.W.); neid_fengweim@163.com (W.M.); qiugrass@126.com (L.Q.); xukemi@bit.edu.cn (K.X.)

**Keywords:** silicon carbide, femtosecond laser processing, phase transformation

## Abstract

Silicon carbide (SiC) is widely used in many research fields because of its excellent properties. The femtosecond laser has been proven to be an effective method for achieving high-quality and high-efficiency SiC micromachining. In this article, the ablation mechanism irradiated on different surfaces of 6H-SiC by a single pulse under different energies was investigated. The changes in material elements and the geometric spatial distribution of the ablation pit were analyzed using micro-Raman spectroscopy, Energy Dispersive Spectrum (EDS), and an optical microscope, respectively. Moreover, the thresholds for structural transformation and modification zones of 6H-SiC on different surfaces were calculated based on the diameter of the ablation pits created by a femtosecond laser at different single-pulse energies. Experimental results show that the transformation thresholds of the Si surface and the C surface are 5.60 J/cm^2^ and 6.40 J/cm^2^, corresponding to the modification thresholds of 2.26 J/cm^2^ and 2.42 J/cm^2^, respectively. The Raman and EDS results reveal that there are no phase transformations or material changes on different surfaces of 6H-SiC at low energy, however, decomposition and oxidation occur and then accumulate into dense new phase material under high-energy laser irradiation. We found that the distribution of structural phase transformation is uneven from the center of the spot to the edge. The content of this research reveals the internal evolution mechanism of high-quality laser processing of hard material 6H-SiC. We expect that this research will contribute to the further development of SiC-based MEMS devices.

## 1. Introduction

Due to their excellent photoelectric properties and physicochemical stability, SiC devices have been widely used in many research fields, such as engine turbines [1], sensors [2,3,4], accelerometers [5,6], electronic circuits [7,8,9,10], biomedicine [11], and thermal piezoresistive devices [12,13]. By designing and processing various micro-fine and micro-nanostructures on SiC wafers, researchers have realized functional applications that are not available in conventional Si-based microelectromechanical systems (MEMS) devices. However, the high stability and hardness of SiC make it difficult to process, which thereby hinders the further development of SiC-based MEMS devices.

Currently, the main processing methods for SiC mainly include mechanical grinding [14], wet etching [15,16], and dry etching [17,18,19,20,21,22]. However, mechanical gear grinding can make SiC prone to edge collapse and fracture damage, which affects machining quality and also causes irreversible damage to the gear. In particular, it is worth noting that the isotropic corrosion characteristics of wet etching cannot realize the preparation of specific micro-structure functional devices. As for the dry etching approach, its etching rate is too low for practical engineering applications. Tightly focused femtosecond laser has been extensively applied for deep-etch patterning of hard materials such as SiC, due to its outstanding advantages of small thermal damage area, high efficiency, and ultra-high mask volume processing.

Nevertheless, it causes the modification of the surface refractive index and a change in the material phase structure when the femtosecond laser irradiates the sample. By controlling the specific parameters of femtosecond laser irradiation, self-assembled periodic nanostructures [23] and SiC optical waveguides [24,25] induced by femtosecond laser irradiation were observed. With the increase in laser fluence, the irradiated sample is melted and reshaped by laser ablation, forming micro-cracks and holes. Feng et al. analyzed the recasting behavior caused by 4H-SiC laser ablation through simulation and experiments and found that there are four forces—including recoil pressure, surface tension, buoyancy, and gravity—in the ablation process, among which recoil pressure and surface tension played the dominant role in the recasting process [26]. Vanthanh et al. successfully prepared holes in 6H-SiC samples with a thickness of 350 μm by selective etching in a mixture of hydrofluoric acid and nitric acid after modification by femtosecond laser irradiation [27]. Furthermore, the effects of pulse number and pulse energy on hole depth and diameter were also analyzed. Xie et al. used a sapphire femtosecond laser with a wavelength of 800 nm and a pulse width of 35 fs to induce ablation in a large-area periodic structure on 4H-SiC; systematically studied the effects of pulse energy, scanning speed, and polarization direction on the morphology and periodicity of the microstructures; and successfully fabricated a planar optical attenuator that realized linearly polarized light [28]. Huang et al. investigated the material rapid etching of single-crystal 6H-SiC by combining femtosecond laser irradiation modification and inductively coupled plasma (ICP) etching, showing that the silicon dioxide and rough surface produced after femtosecond laser irradiation can accelerate the rapid etching of SiC compared to untreated 6H-SiC [29]. By configuring a femtosecond laser with a wavelength of 780 nm into a double-pulse emission device, Kim et al. successfully achieved exfoliation of a 4H-SiC single-crystal wafer with a thickness of 400 μm, and the results showed that the root mean square roughness of the peel surface was 5 μm and the cutting loss thickness was less than 24 μm [30]. Although femtosecond laser processing of SiC has been extensively studied, unfortunately, the internal mechanism of the interaction between the femtosecond laser and SiC has not been fully understood, and its further applications remain to be explored.

Indeed, the intricate internal mechanisms involved in processing are pivotal for achieving high-quality processing and advancing the development of SiC. This paper presents the results of single-pulse irradiation experiments conducted on the Si and C surfaces of 6H-SiC samples with varying pulse energies, followed by a comparative analysis. Meanwhile, qualitative analysis of the surface morphology distributions is performed using optical microscopy. The ablation thresholds of the modification zone and the structural transformation zone are calculated based on ablation theory. The laser-induced material transformations and compositional changes are analyzed by micro-Raman spectroscopy and EDS. In addition, the morphology of the Si and C surfaces is slightly changed at lower energies, but no new phases are formed. At high energies, the laser-induced high temperatures and stress waves cause the Si-C crystal bonds to break, leading to surface melting, decomposition, formation of modification zones and structural transformation zones, and the generation of new amorphous and crystalline silicon phases. By studying the irradiation mechanism, we can realize the preparation of specific functional devices by controlling the specific parameters of femtosecond laser irradiation, which can be widely used in the field of high-precision optoelectronic devices.

## 2. Materials and Methods

### 2.1. Materials Preparation

A 430 μm thick n-type 6H-SiC wafer (Orientation: [0001] ± 0.5°, Resistivity: 0.02–0.1 Ω cm, Ra ≤ 0.2 nm) was adopted in the experiments. The diameter of the experimental sample was 2 inches (Powerway Wafer, Xiamen, China), which was cut into samples with dimensions of 8 × 8 mm. After analyzing the crystal phase of the SiC, Si and C letters were marked on the Si and C surfaces of the SiC to distinguish them. Before laser irradiation, the 6H-SiC sample was ultrasonically cleaned with anhydrous ethanol and deionized water for 20 min.

### 2.2. Experimental Method and Setup

**Figure 1:**
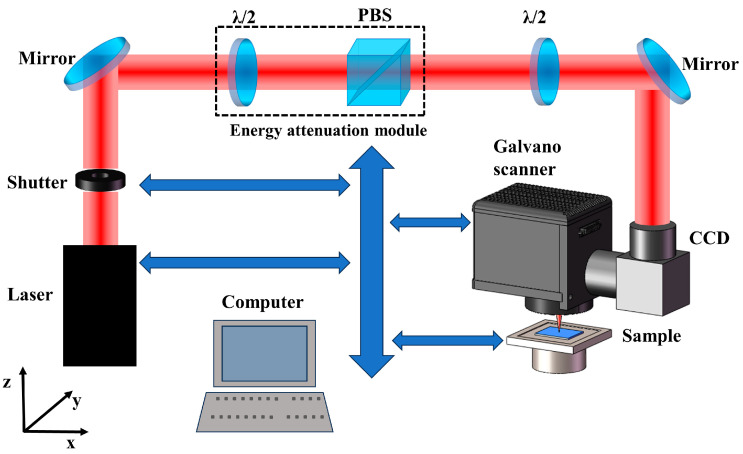
Schematic of experimental setup.

The schematic of the experimental setup is depicted in Figure 1. The femtosecond laser used in this work is a Yb: KGW system (PHAROS, Light Conversion, Vilnius, Lithuania) with a pulse duration of 290 fs, a wavelength of 1030 nm, and a repetition frequency of 50 kHz. A single-pulse laser with Gaussian intensity distribution is delivered to the two-axis galvanometric scanner (Sunny Technology, Beijing, China) and then focused on the sample surface through an F-theta lens (focal length is 100 mm). The galvanometric scanner ensures accurate movement of the focused spot, and the entire machining process can be monitored online by a connected high-speed CCD (Hikvision, Hangzhou, China). During the experiment, the energy can be continuously adjusted through the combination of a zero-order half waveplate and a polarization beam splitter (PBS). The laser polarization can be modulated by a second zero-order half waveplate. The accuracy of the experimental sample’s motion is accomplished by the three-dimensional displacement system (Ludl, New York, NY, USA), which is controlled via high-precision servo motion. To ensure the precision and coordination of the system, all the aforementioned devices communicate with the host computer through serial bus control.

Micro-Raman spectroscopy is a powerful non-destructive testing method. It can obtain molecular vibration or rotation information by detecting and analyzing the displacement, intensity, and peak width of the Raman signal of the sample, and then can identify a variety of information such as material composition, crystallinity analysis, and structure stress with high-resolution [31,32]. In this paper, laser Raman spectroscopy (WITec alpha 300R, Ulm, Germany equipped with a 532 nm laser) was used to obtain the new phase structure distribution and material composition of single-crystal SiC (c-SiC) under different laser irradiation fluences. The surface morphology and distribution of ablation sputtering following laser irradiation with a single pulse were examined and analyzed using a laser confocal microscope (OLS4000, Olympus, Tokyo, Japan). Additionally, the EDS (Apreo S, Thermo Scientific, Waltham, MA, USA) was employed for obtaining the variation in the micro-region element.

## 3. Results

### 3.1. Calculation of Single-Pulse Ablation Threshold

**Figure 2:**
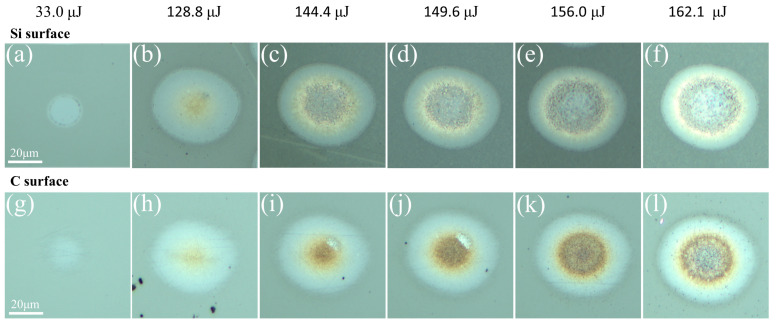
Optical micrograph diagrams of Si and C surfaces of SiC irradiated by laser with single-pulse energy of 33.0 μJ, 128.8 μJ, 144.4 μJ, 149.6 μJ, 156.0 μJ, and 162.1 μJ, respectively. (**a**–**f**) Si surfaces. (**g**–**l**) C surfaces.

To verify the ablation threshold of SiC, a series of single pulses with different energies were applied. Figure 2 shows the optical micrographs of the Si and C surfaces of SiC under laser irradiation with single-pulse energy of 33.0 μJ, 128.8 μJ, 144.4 μJ, 149.6 μJ, 156.0 μJ, and 162.1 μJ, respectively. It can be seen that the Si and C surfaces of SiC are only slightly modified, resulting in refractive index changes at a lower energy of 33.0 μJ. With the increase in energy, a ring-like pattern distribution gradually emerges in the ablation region. Previous research indicates that the central region of the ring pattern signifies the structural transformation area, while the outer region represents the material modification zone [33,34]. Moreover, as the energy levels rise, both the diameter of the modification zone and the structural transformation zone of the material expand. Additionally, it was noted that the ablation effect of the Si surface exceeded that of the C surface at equivalent energy levels. To facilitate a quantitative analysis, we conducted calculations to determine the ablation thresholds for both the Si and C surfaces.

At present, the internal mechanism of femtosecond laser interaction with materials has been sufficiently developed, such as Liu’s ablation threshold theoretical model, which is widely accepted. According to Liu’s ablation theory model [33], we can calculate the single-pulse ablation threshold of SiC. The intensity of the spot focused on the surface of the SiC sample follows Gaussian distribution, and the laser fluence F(r) can be expressed as

(1) $$F(r)=F_{0}\exp(\frac{-2r^{2}}{{r_{0}}^{2}})$$

where $F_{0}$ represents the peak energy density of the focused spot, $r_{0}$ is the beam waist radius of the focused spot, and r represents the distance from the center of the spot to any point. $E_{0}$ is the pulse energy of the focused spot, and the peak energy density $F_{0}$ can be obtained using

(2) $$F_{0}=\frac{2E_{0}}{\pi {r_{0}}^{2}}$$

Based on Liu’s theoretical model of ablation thresholds, this yields

(3) $$D^{2}=2{r_{0}}^{2}\ln(\frac{F_{0}}{F_{th}})$$

where D represents the diameter of the ablation pit, and $F_{th}$ represents the ablation threshold. Therefore, the relationship between the diameter of the ablation pit and the ablation threshold can be obtained from the Equations (1)–(3), as follows:

(4) $$D^{2}=2{r_{0}}^{2}(\ln E_{0}-\ln\frac{\pi {r_{0}}^{2}F_{th}}{2})$$

Therefore, the mathematical model between the ablation diameter and the ablation threshold in the structural modification zone and the structural transformation zone of the Si and C surfaces can be obtained as follows:

(5) $$\begin{array}{l} {D_{m\_si}}^{2}=2{r_{0}}^{2}(\ln E_{0}-\ln\frac{\pi {r_{0}}^{2}F_{th\_m\_si}}{2})\\ {D_{s\_si}}^{2}=2{r_{0}}^{2}(\ln E_{0}-\ln\frac{\pi {r_{0}}^{2}F_{th\_s\_si}}{2})\\ {D_{m\_c}}^{2}=2{r_{0}}^{2}(\ln E_{0}-\ln\frac{\pi {r_{0}}^{2}F_{th\_m\_c}}{2})\\ {D_{s\_c}}^{2}=2{r_{0}}^{2}(\ln E_{0}-\ln\frac{\pi {r_{0}}^{2}F_{th\_s\_c}}{2}) \end{array}$$

Here, $D_{m\_si}$ and $D_{s\_si}$, $D_{m\_c}$ and $D_{s\_c}$ are the ablation pit diameters of the modification zone and the structural transformation zone of the Si and C surfaces. $F_{th\_m\_si}$ and $F_{th\_s\_si}$, $F_{th\_m\_c}$ and $F_{th\_s\_c}$ are the ablation thresholds of the modification zone and the structural transformation zone of the Si and C surfaces.

**Figure 3:**
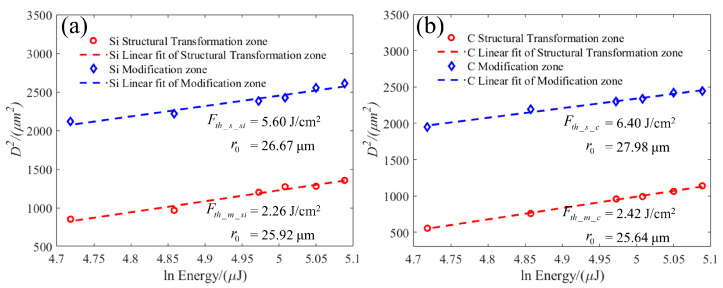
Plot of the spot diameters via the laser pulse energies. (**a**) Si face. (**b**) C face.

The diameter of the laser ablation pit can be accurately measured using confocal microscopy. The results obtained by substituting the above mathematical model are shown in Figure 3. By calculating the intercept of the linear fitting curve on the horizontal axis, we can determine that the ablation thresholds of the modification zone and the structural transformation zone of the Si surface are 2.26 J/cm^2^ and 5.60 J/cm^2^, respectively. Additionally, the waist radii are 25.92 μm and 26.67 μm, as obtained by the linear fitting curve slope. Conversely, the ablation thresholds of the modification zone and the structural transformation zone of the C surface are 2.42 J/cm^2^ and 6.40 J/cm^2^, and the waist radii are 25.64 μm and 27.98 μm, respectively. These findings indicate that the ablation threshold of the Si surface is lower than that of the C surface, indicating that the Si surface is more prone to ablation than the C surface under similar conditions.

Figure 2a,b and Figure 2g,h shows the modified zone and the structural transformation zone of the Si surface and the C surface when the single-pulse radiation energy is 33.0 μJ and 128.1 μJ, respectively. Hence, the thresholds were calculated at 3.231 J/cm^2^ and 12.54 J/cm^2^, respectively. In fact, as the number of irradiation pulses increases, the sample’s ablation threshold will decrease even further until it reaches a saturation state. This phenomenon was observed in a relevant study conducted by Wang et al. [35,36].

### 3.2. Structural Transformation under Different Energies

**Figure 4:**
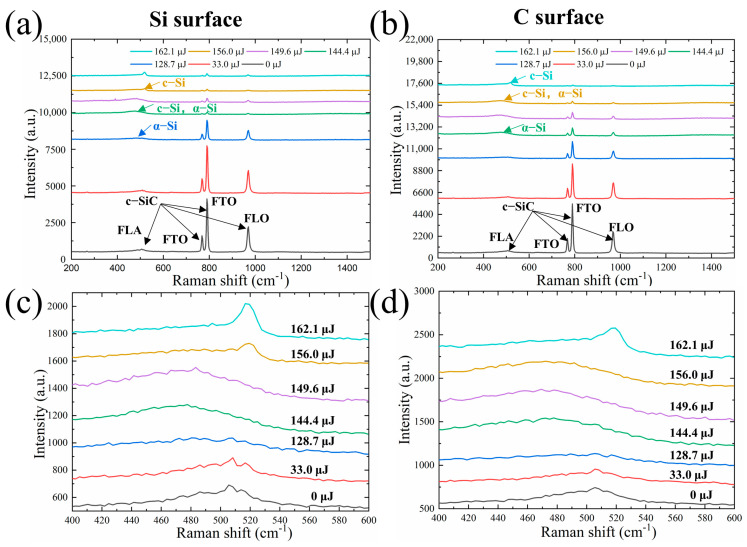
Raman spectrum of SiC irradiated with different laser energies. (**a**,**c**) Raman spectra of Si surface irradiated with different energies. (**b**,**d**) Raman spectra of C surface irradiated with different energies.

To further investigate the evolving interaction mechanism between the laser and the sample, we obtained the Raman spectrum of the pit center following single-pulse ablation of 6H-SiC, as shown in Figure 4. The Si surface Raman spectrum of 6H-SiC is illustrated in Figure 4a; the Raman characteristic peaks of 507 cm^−1^ (FLA), 767 cm^−1^ (FTO), 789 cm^−1^ (FTO), and 967 cm^−1^ (FLO) obtained without laser irradiation (0 μJ) are all Raman peaks generated by optical and acoustic phonons in single-crystalline 6H-SiC, which are consistent with the data in the reference literature [29]. When the single-pulse energy is 33.0 μJ, the peak of the Raman spectrum obtained is the same as that of the unirradiated single-crystal 6H-SiC, but the intensity of the characteristic peak is slightly diminished, indicating that no structural phase transformation to produce new materials occurs at the current energy. The modification mechanism of the surface under the laser-irradiated region compared to the unirradiated region is due to the stress waves generated by the laser on the SiC surface, which are not sufficient to break the tightly bonded Si-C crystal bonds inside the SiC at low energy, so the sample surface is only slightly modified, resulting in physical morphological changes and no new crystal phase formation.

It can be found that the Raman spectrum peak of 507 cm^−1^ is broadened at an energy of 128.7 μJ, and a new amorphous Si phase (480 cm^−1^) appears. However, the intensity of the new spectrum peak (amorphous Si) is weaker than that of the single-crystal 6H-SiC, indicating the predominant retention of the single-crystal 6H-SiC structure within the overall ablated structure. The Raman spectrum peak at 400–600 cm^−1^ was locally amplified to reveal this phenomenon, as shown in Figure 4c. At an energy of 144.4 μJ, the amorphous Si phase (480 cm^−1^) is partially transformed into the single-crystal Si phase (520 cm^−1^), leading to the formation of a mixture of single-crystal Si phase and amorphous Si phase within the ablation region. The Raman intensity of the Si phase surpasses that of the single-crystal 6H-SiC. Subsequently, the center of the ablation structure undergoes complete phase transformation into stable single-crystal Si at an energy of 156.0 μJ. This transformation occurs as a result of increasing energy levels, leading to the breaking of Si-C bonds and the formation of disordered local Si and C clusters distributed throughout the sample. The ultra-short pulse time, limited spatial scale constraints, and the high temperature and pressure in the local space contribute to the transformation of metastable amorphous Si into stable single-crystal Si.

Analysis of the Raman spectrum of the C surface of 6H-SiC in Figure 4b,d indicates that the decomposition of the C surface into single-crystalline Si and amorphous Si exhibits a trend consistent with that observed on the Si surface as energy increases. However, there is a distinction in that the energy threshold for the conversion of SiC into the amorphous Si phase is 144.4 μJ. The minimal presence of the amorphous Si phase at the current energy level, as indicated by the Raman peak, suggests that the structure of the ablated region is predominantly composed of single-crystal SiC. At an energy of 162.1 μJ, the transformation of single-crystal SiC into the amorphous Si phase, followed by its conversion into single-crystal Si, is observed.

**Figure 5:**
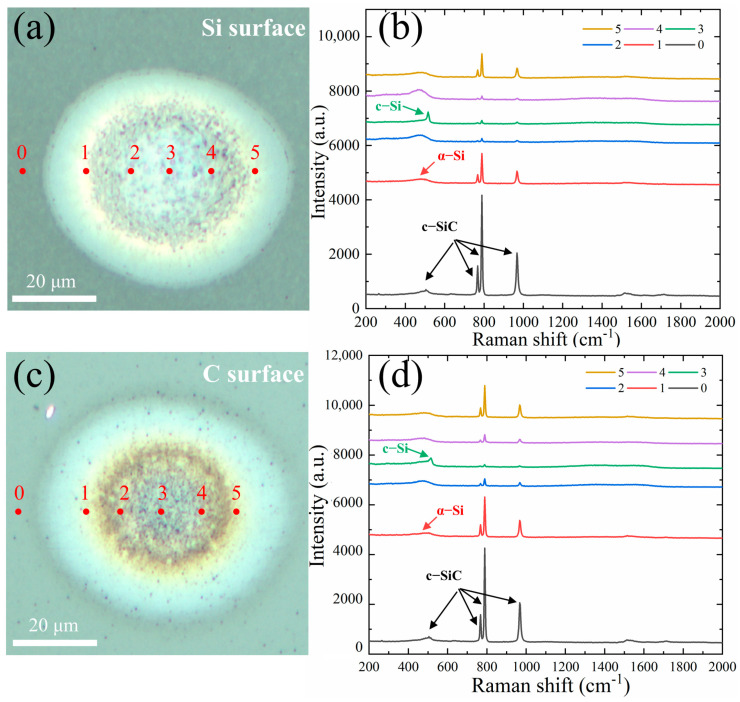
Raman spectrum of different positions in the irradiated region of SiC at an energy of 162.1 μJ. (**a**,**b**) Si face. (**c**,**d**) C face.

Since the intensity of the spot focused on the surface of the sample has a Gaussian distribution, the structural transformation of the laser-irradiated region from the center to the edge of the structure was quantified on different SiC surfaces at an energy of 162.1 μJ, position 0 of the unirradiated region is used as a reference, and the remaining positions (1–5) are evenly distributed along the irradiated region, as shown in Figure 5. Raman spectrum peaks at different positions in the irradiated region of the Si surface are shown in Figure 5a,b. Compared to the unirradiated region at position 0, the Raman peak at the boundary positions 1 and 5 of the irradiated region is still dominated by single-crystal SiC, but the Raman intensity is slightly weakened. As the laser irradiation causes the stacking and dislocation of the SiC structure, the spectrum peak at 507 cm^−1^ is broadened, and an amorphous Si phase (480 cm^−1^) is formed. At positions 2 and 4 of the irradiated region, the higher irradiation energy causes the decomposition of single-crystal SiC to produce more metastable amorphous Si phase content, and the amorphous Si phase material is mainly distributed in the irradiation region.

In central position 3, where the irradiation energy is highest, the SiC is ablated, sprayed, and recrystallized to form a stable single-crystal Si phase (520 cm^−1^) due to the higher temperature and pressure. This can be seen from the sharp c-si (520 cm^−1^) peak that appears in the Raman peak. In addition, it can be seen that the trend of the Raman spectrum peaks at positions 1 and 5, and 2 and 4 are almost the same, indicating that the irradiated energy is the same, which is also consistent with the Gaussian distribution of the irradiated spot intensity. The irradiated region obtained under the above conditions shows a ring distribution, the outer ring being called the modification region and the inner ring being called the structural transformation region.

The Raman spectrum peaks at different positions in the irradiated region of the C surface are shown in Figure 5c,d. The spatial distribution of the irradiated area corresponds to that of the Si face, with the exception that positions 2 and 4 exhibit lower amorphous Si content due to single-crystal SiC decomposition. The predominant structure within the irradiated area is still that of single-crystal SiC.

**Figure 6:**
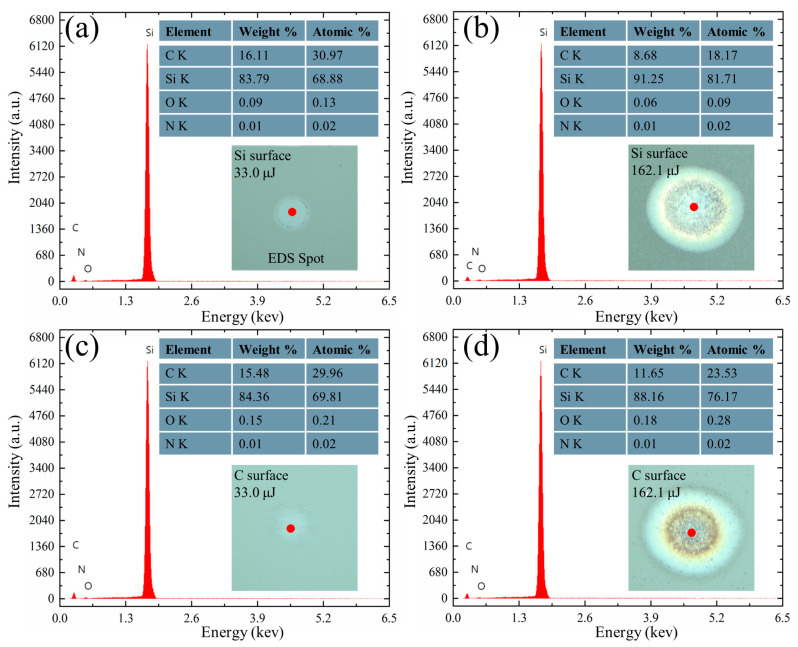
Element distribution on Si and C surfaces under different irradiation energies. (**a**,**b**) Element distribution on the Si surface at energies of 33.0 μJ and 162.1 μJ, respectively. (**c**,**d**) Element distribution on the C surface at energies of 33.0 μJ and 162.1 μJ, respectively.

Energy dispersive spectrum (EDS) was used to analyze the elemental changes in the ablated region. At energies of 33.0 μJ and 162.1 μJ, respectively, the central regions of the ablated structures on the Si and C surfaces were scanned by the EDS. The results are shown in Figure 6. The analysis indicates that Si, C, and O elements are the primary constituents of the irradiated region. The compositional distribution of the ablated region on the Si surface is shown in Figure 6a,b. Si and C elements are the main elements in the ablated region under a low energy of 33.0 μJ irradiation. As the energy level increases to 162.1 μJ, the Si element content gradually increases, while the C element content decreases. Simultaneously, the O element content remains nearly unchanged. This is because single-crystal SiC undergoes almost no structural changes or formation of new phase materials under low-energy laser irradiation; whereas under high-energy irradiation, SiC will explode into Si and C vapor and melt particles at high temperature and pressure, and the Si and C vapor will react with O elements in the air environment to form Si and C oxides. Si and O elements react and eventually accumulate on the surface of the material as Si oxides, and C and O react to produce CO and CO_2_. Therefore, the percentage of Si elements increase and the percentage of C elements decrease [37,38].

The compositional distribution of the ablated region on the C surface is shown in Figure 6c,d. It is evident that the trend of Si, C, and O element content is the same as that of the Si surface during irradiation from low to high energy. However, under the same energy irradiation conditions, the increase in Si elements on the Si surface is higher than that on the C surface, while the decrease in C elements is lower than that on the C surface, which reaffirms that ablation on the Si surface is more intense than that on the C surface.

According to the above experimental results, it is extremely important to understand the internal mechanism behind this. Transparent materials will produce nonlinear absorption and ionization when irradiated by an ultrafast laser, which can promote electrons from the valence band to the conduction band and deposit laser energy into the material, causing permanent damage. There are two main mechanisms by which nonlinear ionization produces electrons: multiphoton ionization (MPI) and avalanche ionization [36,39]. The main mechanisms of ionization to produce electrons can be calculated by the Keldysh parameter [39]. At an energy of 33.0 μJ, the calculated Keldysh parameter was 1.168, indicating that the electrons’ ionization state was mainly tunneling ionization. At 161.2 μJ, the Keldysh parameter was 0.526 and tunneling ionization prevails. After electron ionization, a few free electrons with low kinetic energy will absorb light energy in the form of seed electrons [40], and when their kinetic energy exceeds the band gap width, they collide with valence band electrons, resulting in two low-kinetic-energy conduction-band electrons. This continuous process is called avalanche ionization. In this process, energy is transferred to the SiC lattice through phonons and impurities, resulting in high temperature and high pressure, which causes the SiC to decompose into different phase substances, and the decomposition rate increases linearly with the increase in laser fluence [41]. There will be melting and resolidification around the laser irradiation spot, resulting in the accumulation of oxidized material around the ablative pit, and the whole process can achieve effective material removal.

## 4. Conclusions

This paper investigates the internal ablation mechanism of SiC surfaces under different single-pulse irradiations. The geometric spatial distribution of the ablation pits was observed and analyzed by optical microscopy. The modification thresholds and structural transformation thresholds of different surfaces were calculated according to Liu’s theory. Furthermore, the changes in the elements of the material in the laser ablation pit were observed and analyzed by means of micro-Raman spectroscopy and EDS. The measured results indicate that the structural transformation and modification thresholds of the Si surface are 5.60 J/cm^2^ and 2.26 J/cm^2^, whereas the structural transformation and modification thresholds of the C surface are 6.40 J/cm^2^ and 2.42 J/cm^2^, respectively. This indicates that the Si surface is more prone to ablation compared to the C surface under the same processing conditions. Results of the Raman spectrum and EDS show that there are no structural or new phase material changes on different surfaces of SiC at lower energy levels. However, under high-energy laser irradiation, the Si-C bonds of single-crystal SiC (c-SiC) begin to break and decompose to form new Si phase material, and the high temperature and high pressure induce oxidative stacking of silicon and the formation of dense oxidized nanostructures. This research is significant in revealing the high-quality laser processing mechanism of the hard material 6H-SiC.

## Data Availability

Data are contained within the article.
